# Rodent fibroblast model for studies of response of malignant cells to exogenous 5-aminolevulinic acid

**DOI:** 10.1038/sj.bjc.6690409

**Published:** 1999-05

**Authors:** G Li, M R Szewczuk, L Raptis, J G Johnson, G E Weagle, R H Pottier, J C Kennedy

**Affiliations:** Departments of Microbiology and Immunology, Medicine, Pathology, Oncology, Queen's University, Kingston, Ontario, K7L 3N6, Canada; Department of Chemistry and Chemical Engineering, The Royal Military College of Canada, Kingston, Ontario, K7K 5L0, Canada

**Keywords:** photodynamic therapy, aminolevulinic acid, protoporphyrin, malignant transformation, fibroblasts, oncogenes

## Abstract

All nucleated mammalian cells synthesize protoporphyrin IX (PpIX) when exposed to exogenous 5-aminolevulinic acid (ALA). The response to exogenous ALA under standard conditions (the ALA phenotype) is characteristic for each cell type. Significantly more PpIX accumulates in malignant and premalignant cells than in the normal cells from which they were derived. A rodent fibroblast model was developed to study the mechanisms responsible for this phenomenon. Exogenous ALA induced the accumulation of substantial concentrations of PpIX in fibrosarcoma cells, and in immortalized fibroblasts transfected with the oncogene c-*myc*, IGF-1 receptor, IGF-1 and its receptor, v-*fos*, v-*raf*, v-Ki-*ras*, v-*abl*, or polyomavirus middle T antigen with G418 resistance selection. Much lower concentrations of PpIX accumulated in primary fibroblast cultures, in immortalized fibroblast cell lines, and in immortalized fibroblasts transfected with the G418-resistance gene only. The mechanisms responsible for the increased accumulation of ALA-induced PpIX by transformed cells (the malignant ALA phenotype) therefore appear to be closely linked to the mechanisms responsible for malignant transformation. Identification of the nature of that linkage may lead to new approaches to cancer therapy. © 1999 Cancer Research Campaign


*British Journal of Cancer* (1999) **80**(5/6), 676–684
© 1999 Cancer Research Campaign
Article no. bjoc.1998.0409

### Rodent fibroblast model for studies of response of malignant cells to exogenous 5-aminolevulinic acid

**G Li[1], MR Szewczuk[1,2], L Raptis[1,3], JG Johnson[3], GE Weagle[5], RH Pottier[5] and JC Kennedy[3,4,5]**

Departments of [1]Microbiology and Immunology, [2]Medicine, [3]Pathology and [4]Oncology, Queen's University, Kingston, Ontario K7L 3N6, Canada; [5]Department of Chemistry and Chemical Engineering, The Royal Military College of Canada, Kingston, Ontario K7K 5L0, Canada

**Summary** All nucleated mammalian cells synthesize protoporphyrin IX (PpIX) when exposed to exogenous 5-aminolevulinic acid (ALA). The response to exogenous ALA under standard conditions (the ALA phenotype) is characteristic for each cell type. Significantly more PpIX accumulates in malignant and premalignant cells than in the normal cells from which they were derived. A rodent fibroblast model was developed to study the mechanisms responsible for this phenomenon. Exogenous ALA induced the accumulation of substantial concentrations of PpIX in fibrosarcoma cells, and in immortalized fibroblasts transfected with the oncogene c-*myc*, IGF-1 receptor, IGF-1 and its receptor, v-*fos*, v-*raf*, v-Ki-*ras*, v-*abl*, or polyomavirus middle T antigen with G418 resistance selection. Much lower concentrations of PpIX accumulated in primary fibroblast cultures, in immortalized fibroblast cell lines, and in immortalized fibroblasts transfected with the G418-resistance gene only. The mechanisms responsible for the increased accumulation of ALA-induced PpIX by transformed cells (the malignant ALA phenotype) therefore appear to be closely linked to the mechanisms responsible for malignant transformation. Identification of the nature of that linkage may lead to new approaches to cancer therapy.

**Keywords:** photodynamic therapy; aminolevulinic acid; protoporphyrin; malignant transformation; fibroblasts; oncogenes

Most nucleated mammalian cells respond to exogenous 5-aminolevulinic acid (ALA) by synthesizing at least small quantities of protoporphyrin IX (PpIX). Since PpIX is a highly fluorescent photosensitizer, cells that synthesize and accumulate relatively high concentrations of PpIX may be identified by their PpIX fluorescence and destroyed by exposure to photoactivating light. Most malignant and at least some premalignant cells accumulate substantially more ALA-induced PpIX than do the normal cells from which they arose. This difference in their response to exogenous ALA (their ALA phenotype) is so consistent that it has been used clinically for the detection of malignant tissues by ALA-induced fluorescence and for the selective phototoxic destruction of malignant cells by ALA-induced photosensitization (Kennedy et al, 1990, 1996; Kennedy and Pottier, 1992; Peng et al, 1997*a*, 1997*b*). The clinical observation that the malignant form of the ALA phenotype is expressed by at least some premalignant tissues suggests that the altered response to ALA may reflect some basic biochemical difference between normal and malignant or premalignant cells which appears quite early during the process of malignant transformation. Such a difference might be exploited therapeutically (Campbell et al, 1996*b*). However, neither the mutation(s) ultimately responsible for the increased accumulation of ALA-induced PpIX nor the mechanisms immediately responsible for the observed alteration in haem biosynthesis during the process of malignant transformation have yet been identified.

*Received 9 July 1998*
*Revised 2 December 1998*
*Accepted 3 December 1998*

*Correspondence to:* G Li, Kingston Regional Cancer Centre, 25 King Street W, Kingston, Ontario K7L 5P9, Canada

The rodent fibroblast model described below was developed to study biochemical and genetic changes associated with development of the malignant ALA phenotype during the process of transformation. The response to exogenous ALA of primary cultures of embryonic mouse fibroblasts was compared with that of established lines of fibrosarcoma cells (positive control), immortalized but non-malignant fibroblasts (immortalization control), immortalized fibroblasts transfected with selected non-oncogenes (transfection control) and immortalized fibroblasts transfected with well-known oncogenes whose corresponding oncoproteins influence the signal transduction pathway at sites ranging from the outer plasma membrane to the nucleus. Immortalized fibroblasts (normal and oncogene-transfected) from two different strains of mouse and from one strain of rat were used in certain experiments to demonstrate that the responses to exogenous ALA were neither strain- nor species-specific. Two human carcinoma cell lines were included to permit comparison with the response of common human cancers.

### MATERIALS AND METHODS

### Chemicals

5-Aminolevulinic acid hydrochloride (ALA) was purchased from Fluka Chemie AG, Buchs, Switzerland, and used without further purification.

### Mice

Female Balb/c mice were purchased from Charles River Canadian Breeding Farm and Laboratories, Montreal, Quebec, Canada, and maintained in the Animal Care Facilities of Queen's University in accordance with Canadian Council for Animal Care standards. Procedures were subject to approval by the University Animal Care Committee.

### Cells and cell culture

Primary cultures of mouse embryonic fibroblasts (MEF) were obtained by explanting cells from 14- to 16-day-old fetuses (Loo and Cotman, 1998). Immortalized fibroblast cell lines (Balb/c 3T3, NIH 3T3 and Swiss 3T3), Balb/c mouse fibrosarcoma cell lines (Wehi 164, 6418 and 6423), human bladder carcinoma cell line T24 and human lung cancer cell line A549 were purchased from the American Type Culture Collection (ATCC, Rockville, MD, USA). The Fischer rat fibroblast line F-111 and its derivatives PyF-111 (F-111 transformed by polyomavirus infection), mT-2 (F-111 transfected with polyomavirus mT antigen oncogene), Gen-1 (F-111 transfected with polyomavirus early region mT cDNA under the control of the mouse mammary tumour virus MMTV promoter), pymT4d (F-111 transfected with polyoma mT under the control of the polyomavirus promoter), Fneo-1v and Fneo-1w (F-111 cells transfected with the G418-resistance gene alone) were described previously (Raptis et al, 1985). Primary

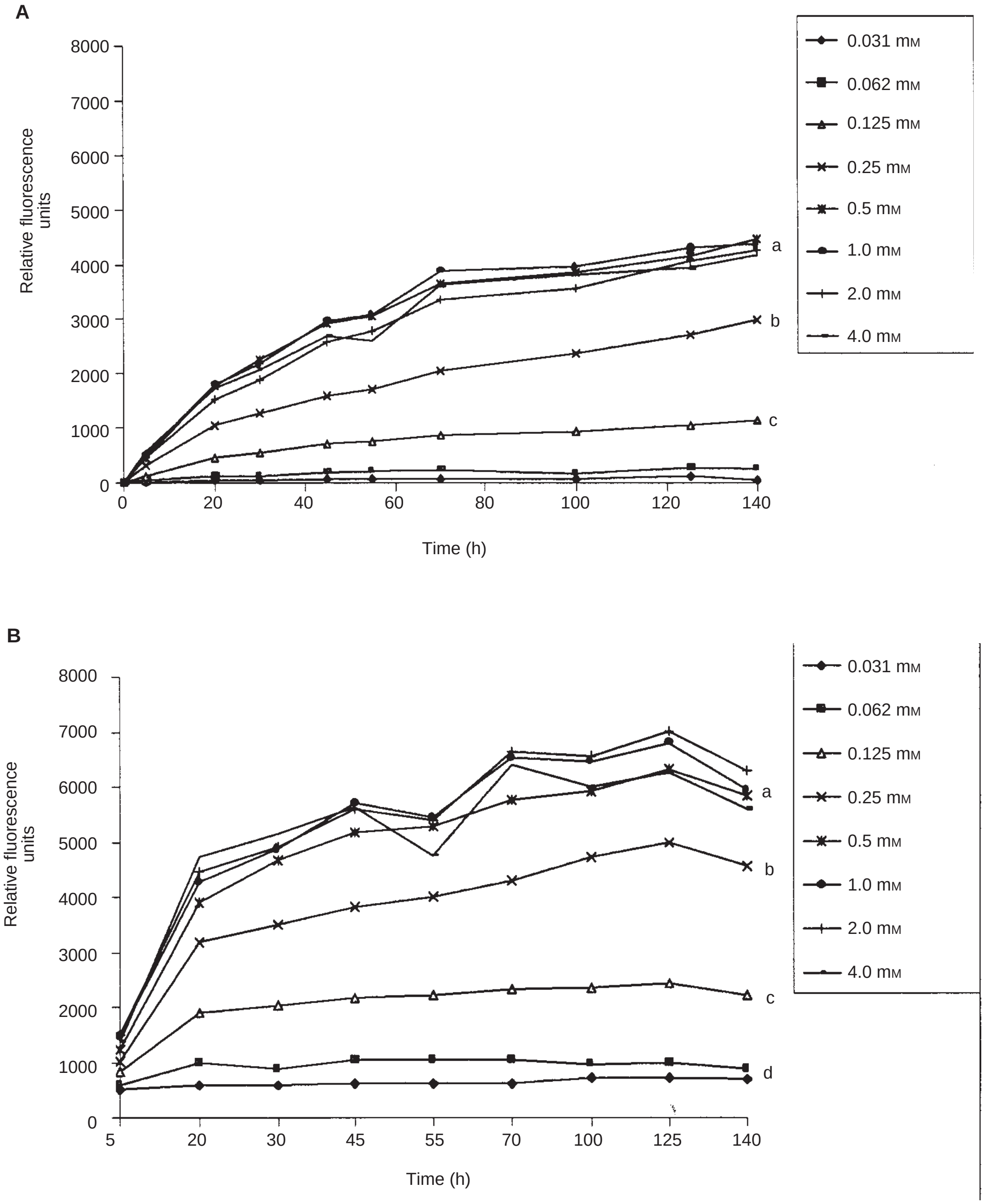

**Figure 1** Effect of ALA concentration and duration of incubation on intensity of PpIX fluorescence in confluent cultures of Balb/c 3T3 fibroblasts (**A**) and Balb/c Wehi 164 fibrosarcoma cells (**B**). PpIX was identified and the fluorescence intensity at 635 nm measured by spectrophotofluorometry (four cultures per data point). Significant differences between curves ($P < 0.05$, Multiple Range Test) are indicated by lower case letters to the right of the curves

*© Cancer Research Campaign 1999*

culture (tertiary passage) Fischer rat fibroblasts transfected with v-*abl* were a gift from Dr T Yamashita (Yamashita et al, 1988). Balb/c 3T3 cells transfected with the oncogene c-*myc* (3T3/c-*myc*), with insulin-like growth factor-1 receptor (3T3/IGF-1 receptor), or with IGF-1 and its receptor (3T3/IGF-1 & receptor) were a gift from Dr R Baserga (Baserga et al, 1992). NIH 3T3 cells transfected with oncogene v-*raf* (3T3/EH v-*raf*), with v-*fos* (3T3/FBR v-*fos*), or with v-Ki-*ras* (3T3/v-Ki-*ras*) were a gift from Dr U Rapp (Kiss et al, 1991).

All cells were maintained at 37°C in 5% carbon dioxide and humidified air. Primary cultures of Balb/c fetal fibroblasts were cultured in Dulbecco's modified Eagles medium (DMEM) (Gibco, Burlington, Ontario, Canada) containing 10% fetal bovine serum (Gibco). Balb/c cell lines 3T3/IGF-1 receptor, 3T3/IGF-1 & receptor, and 3T3/c-*myc* were cultured in DMEM with 5% fetal bovine serum (FBS) plus 5% calf serum, with selection pressure for the transfected gene maintained, respectively, by the addition of 200 μg $ml^{-1}$ G418 (geneticin, Life Technologies, Grand Island, NY, USA), or 200 μg $ml^{-1}$ G418 plus 150 μg $ml^{-1}$ hygromycin (Boehringer Mannheim, Mannheim, Germany), or 400 μg $ml^{-1}$ G418. Wehi 164 was cultured in RPMI-1640 (Baker's X-tra Soluble, Hyclone, Logan, UT, USA) with 10% FBS. Human bladder carcinoma line T24 was cultured in McCoy's 5a Medium (Life Technologies, Grand Island, NY, USA) with 10% FBS. All of the other cell lines were cultured in DMEM with 10% FBS.

Cell lines were passaged three times when first obtained, and then frozen and stored at –80°C or in liquid nitrogen to provide low-passage cells to renew the stock as required. Our policy was to re-establish the line from frozen stock after a maximum of 20 weeks in culture.

### ALA-induced accumulation of PpIX in vitro

Unless otherwise specified, confluent cultures of cells were incubated in the dark for 15 h with 3 mM ALA and 1% FBS in RPMI-1640 without Phenol Red (RPMI). These conditions had been selected as optimal on the basis of the experiments summarized in Figure 1. Other concentrations of ALA and times of incubation were used as required by specific protocols.

### ALA-induced fluorescence quantitated by flow cytometry

The basic technique was reported previously (Campbell et al, 1996*a*). Cultures of fibroblasts were incubated in the dark with exogenous ALA as described above. Single-cell suspensions for flow cytometry were prepared from fibroblast monolayers by washing the cells loose with a jet from a pipette, and then suspending them in a final volume of 0.5 ml RPMI. DNA-Check Beads 5–20 μm in diameter (Coulter Electronics) were used to calibrate and standardize the system and to facilitate measurements of cell size. An EPICS Elite flow cytometer (Coulter Electronics, Burlington, Ontario, Canada) was used with an excitation wavelength of 488 nm (100 mW) and a 630/20 nm bandpass filter for isolation of the PpIX fluorescence.

### ALA-induced PpIX fluorescence quantitated by spectrophotofluorometry

The instrumentation previously described for in vivo studies of fluorescence (Golub et al, 1998) was modified slightly for use in vitro. In brief, quasi-monochromatic 410 nm light was directed onto confluent monolayers of cells in 96-well plates by passing the light through one limb of a bifurcated optical fibre bundle whose common end was directed upward through a small hole in a table over which individual wells in the 96-well plates could be positioned for fluorescence measurements. Since the diameter of the common end of the bifurcated optical fibre bundle closely matched the diameter of the wells, it was possible to illuminate each monolayer culture without cross-talk from adjacent wells. The mixture of scattered and fluorescent light from the cell monolayers was transmitted along the other limb of the bifurcated fibre bundle for analysis. At least ten replicate wells were evaluated for each point, to minimize the effect of possible variations in cell density, although care was taken to ensure that all cultures (freshly explanted, immortalized and transformed) were both confluent and in monolayers. Autofluorescence (no ALA added) was subtracted from all readings. The fluorescence of a standard acidic solution of uroporphyrin was measured at regular intervals during each experiment to detect and compensate for possible fluctuations in sensitivity of the apparatus. Since the technique provided complete emission spectra as well as intensity measurements, it was possible to identify the fluorochrome being measured as PpIX rather than Zn-PpIX, uroporphyrin I or III, or coproporphyrin I or III.

### ALA-induced PpIX quantitated by chemical extraction

A 1:1 mixture of 1.0 N perchloric acid and methanol was used to extract PpIX from both cells and culture supernatants (Roy et al, 1997). Confluent cultures of cells were incubated with 3 mM ALA for periods of time specified in Figure 3, and both cells and supernatants harvested. The cells were counted using trypan blue to assess viability and pelleted by centrifugation. Both cells and supernatants were stored at –80°C in the dark until required. Each cell pellet was mixed with 3 ml of the extraction solution, homogenized mechanically (24 000 rpm for 5–10 s), ultrasonicated in a water bath for 1 h, and then centrifuged at 1700 ***g*** for 10 minutes. The PpIX in the supernatants was extracted by mixing 2.5 ml of the extraction solution with 0.5 ml of supernatant, and then processing as described above. The PpIX was assayed spectrophotometrically (LS100, Photon Technology International, London, Ontario, Canada) by comparison to a standard curve prepared from purified PpIX (Porphyrin Products Inc., Logan, UT, USA) dissolved in Tris-HCL and DMSO (concentration range 1–400 ng $ml^{-1}$) with the excitation wavelength set at 400 nm and fluorescence intensities measured from 550–750 nm.

### Quantitation of ALA-induced photosensitization by $^3$H-thymidine incorporation

Following incubation with 3 mM ALA for 15 h as described above, cells were harvested and counted and the concentrations adjusted so that each cell suspension contained the same number of cells (minimum number $5 \times 10^5$). Aliquots (200 μl) were placed in polystyrene flow cytometry tubes (Falcon #2058, Becton Dickinson, Lincoln Park, NJ, USA), and exposed to graded doses of photoactivating light as described previously (Campbell et al, 1996*a*). The fluence was approximately 70 mW $cm^{-2}$ (Model 210 Power Meter, Coherent Radiation). After an additional 2 h of incubation in the dark to allow at least some of the ongoing DNA synthesis in lethally damaged cells to shut down, 600 μl of medium was added to each cell suspension, which was then subdivided as 200 μl

 © Cancer Research Campaign 1999

aliquots into four wells of a 96-well plate. A total of 25 μl of medium containing 1 μCi of $^3$H-thymidine (Dupont, Montreal, Canada) was added to each well and incubated for an additional 18 h, after which the cells were harvested with an automated multiwell harvester (Skatron, Lierbyen, Norway) that aspirated and lysed the cells, transferred the DNA to a filter paper, and washed away unincorporated $^3$H-thymidine. The filter paper was transferred to a 6 ml scintillation vial (Simport Plastics, Beloeil, Quebec) and 3 ml of Aquamix (ICN Radiochemicals, Irvine, CA, USA) was added. The samples were analysed using a Beckman LF6000LL scintillation counter. Negative controls for each experiment included cells incubated with ALA but not exposed to light, and cells not incubated with ALA but exposed to the highest dose of light used in the experiment.

### Anchorage independence assay

Approximately $5 \times 10^3$ cells were suspended in 2 ml of 0.3% Bacto-Agar (Difco Laboratories, Detroit, MI, USA) in DMEM supplemented with 5% calf and 5% FBS, and overlaid on a base of 0.5% agar in the same medium in 6-well plates. The cells were incubated for 2–4 weeks until colonies were visible to the eye.

### ALA-induced photosensitization of tumours in vivo

Six-week-old female Balb/c mice were injected subcutaneously (s.c.) with 0.2 ml of fibrosarcoma cell suspensions containing $5 \times 10^6$ cells ml$^{-1}$. Mice with tumours approximately 2 mm in diameter were selected for the photosensitization experiments. ALA was injected intraperitoneally (i.p.) into three groups of three mice at doses of 100, 200, or 300 mg ALA per kg of body weight. Three hours after the injection of ALA, each mouse was anaesthetized by an i.p. injection of ketamine (Rogarsetic, Pfizer Canada Animal Health, London, Ontario, Canada) at a dose of 100–120 mg kg$^{-1}$ of body weight and shielded with aluminium foil except for a hole overlying the tumour. The output of a 650 W quartz halogen lamp was passed in succession through a cold water bath containing a 590 nm longpass filter and a Schott KG3 heat filter. Each tumour received 200 J cm$^{-2}$ of 600–800 nm light at 90 mW cm$^{-2}$ from this source. The mice were then returned to the dark and their tumours observed daily.

### Statistical procedures

Fluorescence intensities are presented as mean ± s.d. Statistical analyses were performed with SAS/STAT software on a Unix computer system. Significant differences ($P < 0.05$) detected by the Multiple Range Test (MRT) are indicated by lower case letters to the right of the curves. A two-way analysis of variance (ANOVA) statistical program was used to measure the significance of differences between curves, and Student's *t*-test for differences between selected data points for different cell lines.

### RESULTS

### Cell-associated ALA-induced fluorochrome identified by spectrophotofluorometry

Gen-1 rat fibroblasts were incubated for 15 h with 3 mM ALA as described above, without inducer. Under such conditions, Gen-1 cells express approximately 5% of the levels of mT that are present in F-111 cells transformed by polyomavirus (Raptis et al,

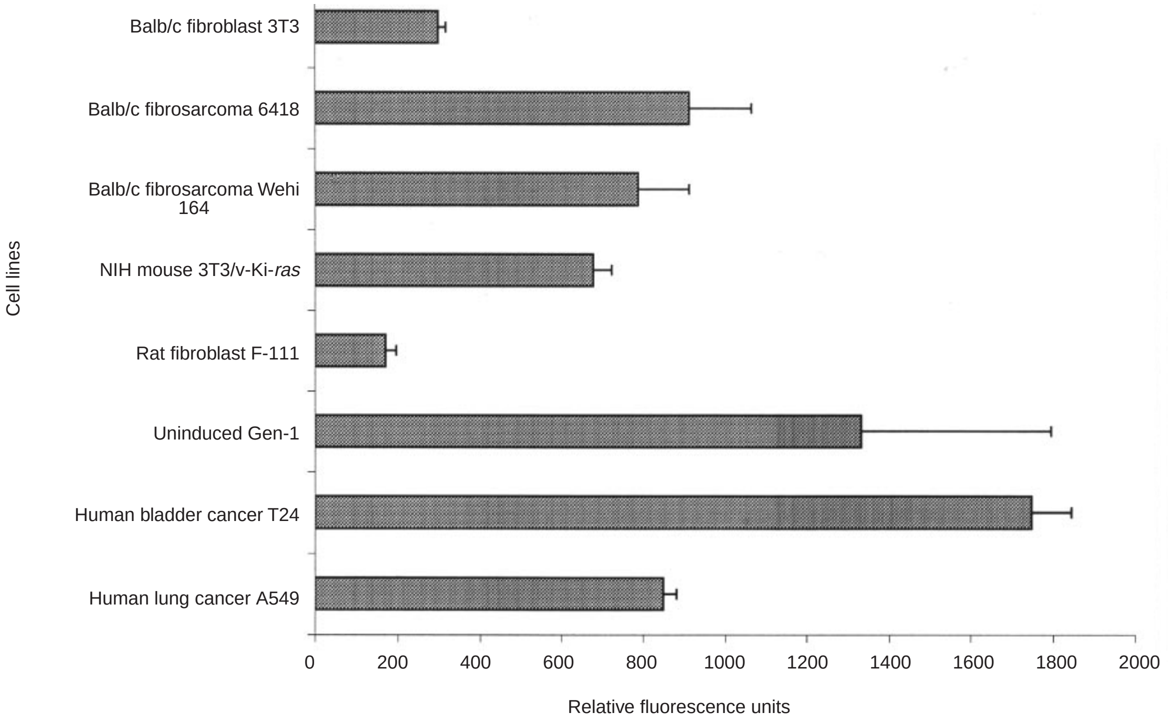

**Figure 2** Spectrophotofluorometric quantitation (mean ± s.d.) of ALA-induced PpIX fluorescence at 635 nm in cultures of immortalized mouse and rat fibroblasts, oncogene-transfected fibroblasts, fibrosarcoma cells and human cancer cell lines. Cells were incubated for 15 h with 3 mM ALA. All differences between immortalized mouse or rat fibroblasts and the other cell lines were significant ($P = 0.03$ or less)

© Cancer Research Campaign 1999 

1985). The emission spectrum of the cell-associated red fluorescence was characteristic of intracellular PpIX, with no fluorescence attributable to uroporphyrin I or III, coproporphyrin I or III, or Zn-protoporphyrin.

### Quantitation of cell-associated ALA-induced PpIX by spectrophotofluorometry

Confluent cultures of Balb/c mouse 3T3 cells and Balb/c mouse fibrosarcoma cell line Wehi 164 were incubated with concentrations of ALA varying from 0 to 4.0 mM, for periods of time ranging from 5 to 140 h. The ALA-induced PpIX fluorescence per standard area of culture dish was measured by spectrophotofluorometry, and the background (no ALA) fluorescence subtracted (Figure 1). Both cell lines developed PpIX fluorescence in a concentration-dependent and time-dependent manner. Low concentrations of ALA induced no detectable fluorescence, but as the concentration of ALA increased from 0.062 to 0.5 mM, the cell-associated fluorescence increased also. Increases in ALA concentration beyond 0.5 mM did not lead to a statistically significant increase in PpIX fluorescence in either the 3T3 or the fibrosarcoma cells. As the duration of exposure to ALA increased, the fibrosarcoma cells showed a relatively rapid increase in fluorescence during the first 20 h. At all time points the fluorescence of the fibrosarcoma cells was greater than that of the 3T3 cells incubated with the same concentration of ALA.

Figure 2 summarizes data from experiments in which the ALA-induced PpIX fluorescence was measured in a similar manner but at a single time point (after 15 h of incubation with 3 mM ALA). Mouse 3T3 cells accumulated significantly less ALA-induced fluorescence than did fibrosarcoma 6418 ($P = 0.01$), fibrosarcoma Wehi 164 ($P = 0.02$), or 3T3 cells transfected with v-Ki-*ras* ($P = 0.02$). Immortalized rat fibroblasts (F-111 cells) responded to exogenous ALA in a similar manner, accumulating much less than F-111 cells transfected with uninduced polyomavirus mT oncogene ($P = 0.001$). Human bladder carcinoma and human lung carcinoma cell lines showed ALA-induced fluorescence intensities comparable to those of the transformed mouse and rat cells. In all

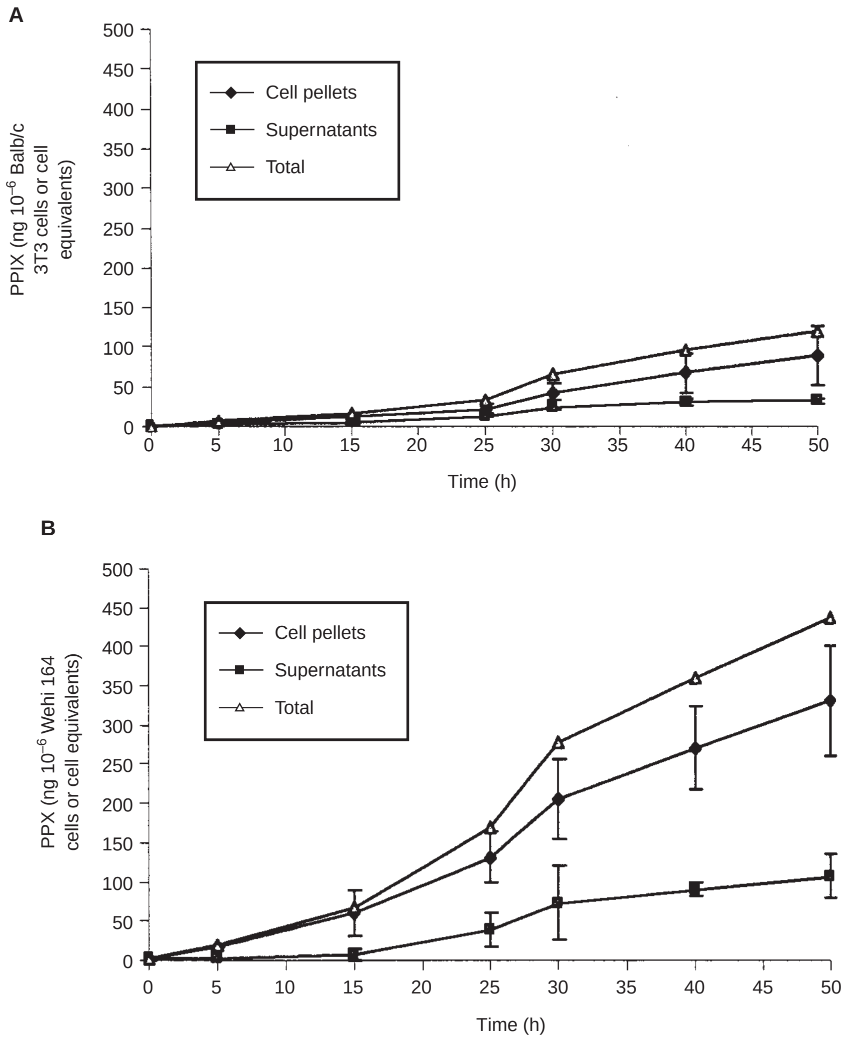

**Figure 3** Spectrophotofluorometric quantitation (mean ± s.d.) of PpIX extracted from cells and supernatants of confluent cultures of Balb/c 3T3 fibroblasts (**A**) and Balb/c Wehi 164 fibrosarcoma cells (**B**) following incubation with 3 mM ALA for specified periods of time. Culture supernatants were measured in 'cell equivalents' by calculating the extracted volume that would have contained exactly $10^6$ cells. Differences between Balb/c 3T3 fibroblasts and Balb/c Wehi 164 fibrosarcoma cells were highly significant ($P < 0.0005$, ANOVA)

© Cancer Research Campaign 1999

cases, the transformed cells were characterized by significantly more ALA-induced PpIX fluorescence than the non-transformed 3T3 and F-111 cells.

### Quantitation of ALA-induced PpIX by chemical extraction

Balb/c 3T3 fibroblasts and Wehi 164 fibrosarcoma cells were incubated with 3 mM ALA in RPMI containing 1% FBS for times up to 50 h. PpIX was extracted from both the washed cells and the culture medium and quantitated by comparison with a standard curve. The time-dependent responses (Figure 3) were comparable to those observed when the ALA-induced PpIX was quantitated by spectrophotofluorometry (Figure 1). Both the immortalized fibroblasts and the fibrosarcoma cells lost PpIX to the culture medium, but for any given time of incubation the fibrosarcoma cells both accumulated and excreted more PpIX than the non-malignant fibroblasts.

### Quantitation of cell-associated ALA-induced PpIX by fluorescence flow cytometry

Normal, immortalized, transfected and malignant mouse and rat fibroblasts were incubated for 15 h in 1% FBS in RPMI with or without 3 mM ALA, and the cell-associated fluorescence at 630 nm then quantitated by flow cytometry. Cells that had been incubated with ALA showed substantially greater fluorescence at 630 nm than those that had not. Freshly explanted Balb/c MEF, immortalized Balb/c mouse fibroblasts (Balb/c 3T3 cells) from two different sources and immortalized NIH mouse fibroblasts (NIH 3T3 cells) from three different sources showed relatively low and statistically indistinguishable levels of ALA-induced fluorescence intensity (Figure 4). The fluorescence of the Balb/c 3T3 cells was significantly less than that of Balb/c fibrosarcomas Wehi 164 ($P = 0.02$) and 6423 ($P = 0.01$), and less also than Balb/c 3T3 cells transfected with c-*myc* ($P = 0.02$), IGF-1 receptor ($P = 0.04$), or IGF-1 & receptor ($P = 0.01$). The fluorescence of the NIH 3T3 cells was likewise less than that of NIH 3T3 cells transfected with v-KI-*ras* ($P = 0.02$), v-*raf* ($P = 0.02$), or v-*fos* ($P = 0.003$). In similar studies using rat fibroblasts (Figure 5), the ALA-induced fluorescence intensities in immortalized Fischer rat fibroblasts (F-111 cells) or in F-111 fibroblasts transfected with the *neo* resistance gene alone were significantly lower than the intensities in rat fibroblasts transformed by infection with polyomavirus ($P = 0.01$) or by transfection with a polyomavirus mT oncogene ($P = 0.001$), with polyomavirus mT early region cDNA ($P = 0.001$), or with either of two different v-*abl* oncogenes ($P = 0.02$, 0.006). The transformed cells (fibrosarcoma, oncogene-transfected, or polyomavirus-transformed) always showed significantly higher levels of ALA-induced fluorescence than the non-neoplastic cells (mouse embryonic fibroblasts, immortalized mouse or rat fibroblasts, or fibroblasts transfected with the non-oncogenic *neo*

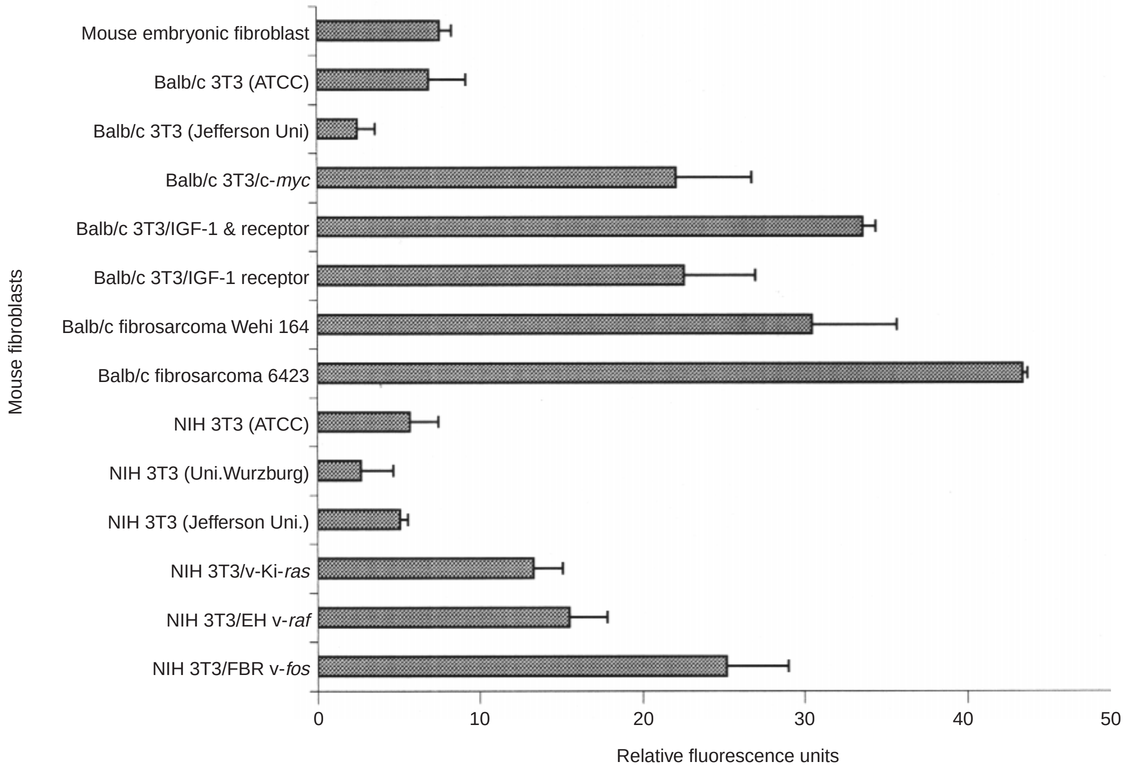

**Figure 4** ALA-induced fluorescence in murine fibroblasts quantitated by flow cytometry (630 nm, mean ± s.d.). Freshly explanted embryonic fibroblasts, immortalized 3T3 fibroblasts, oncogene-transfected 3T3 fibroblasts, and fibrosarcoma cells were incubated for 15 h with 3 mM ALA. Responses of Balb/c embryonic fibroblasts, Balb/c 3T3 cells and NIH 3T3 cells were not significant ($P = 0.2–0.9$, Student's *t*-test). *P*-values for differences between Balb/c 3T3 or NIH 3T3 cells and 3T3 cells transfected with specific oncogenes ranged from 0.04 to 0.003

© Cancer Research Campaign 1999 

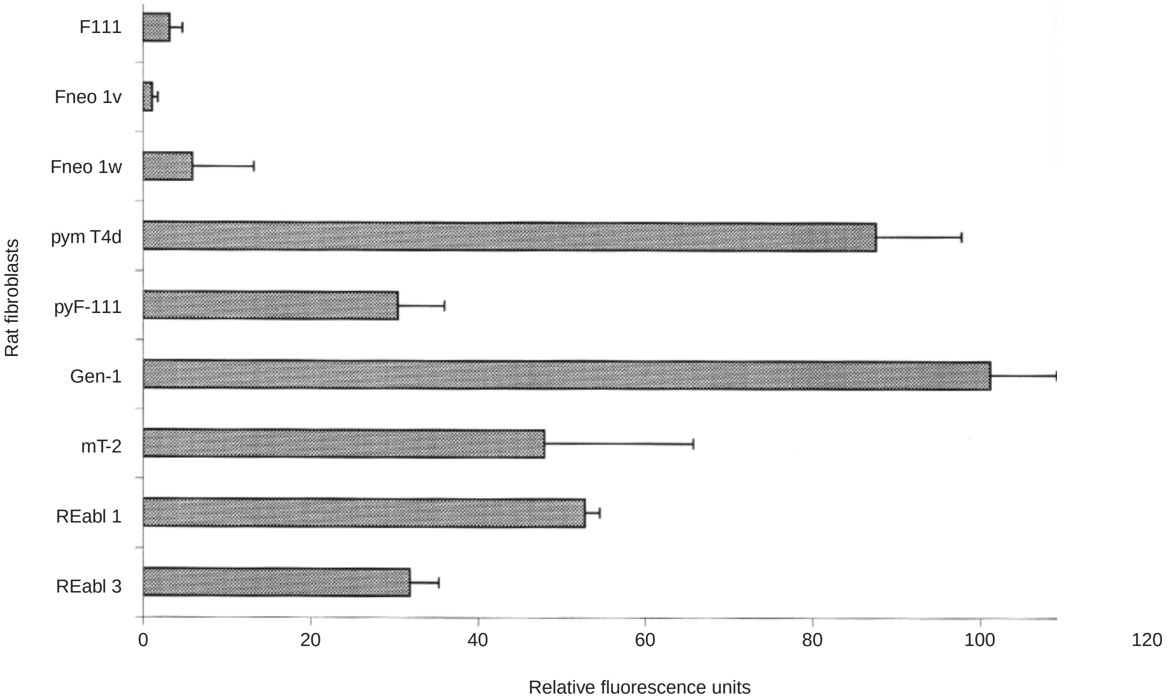

**Figure 5** ALA-induced fluorescence in rat fibroblasts quantitated by flow cytometry (630 nm, mean ± s.d.). Cells had been incubated for 15 h with 3 mM ALA without added inducer. Differences between immortalized Fischer rat fibroblasts (F-111 cells) and F-111 cells transfected with the G418-resistance gene were not significant ($P$ = 0.1–0.2, Student's *t*-test). *P*-values for differences between F-111 cells and immortalized Fischer rat fibroblasts transformed by infection with polyomavirus (pyF-111) or transfected with specific oncogenes ranged from 0.05 to 0.001

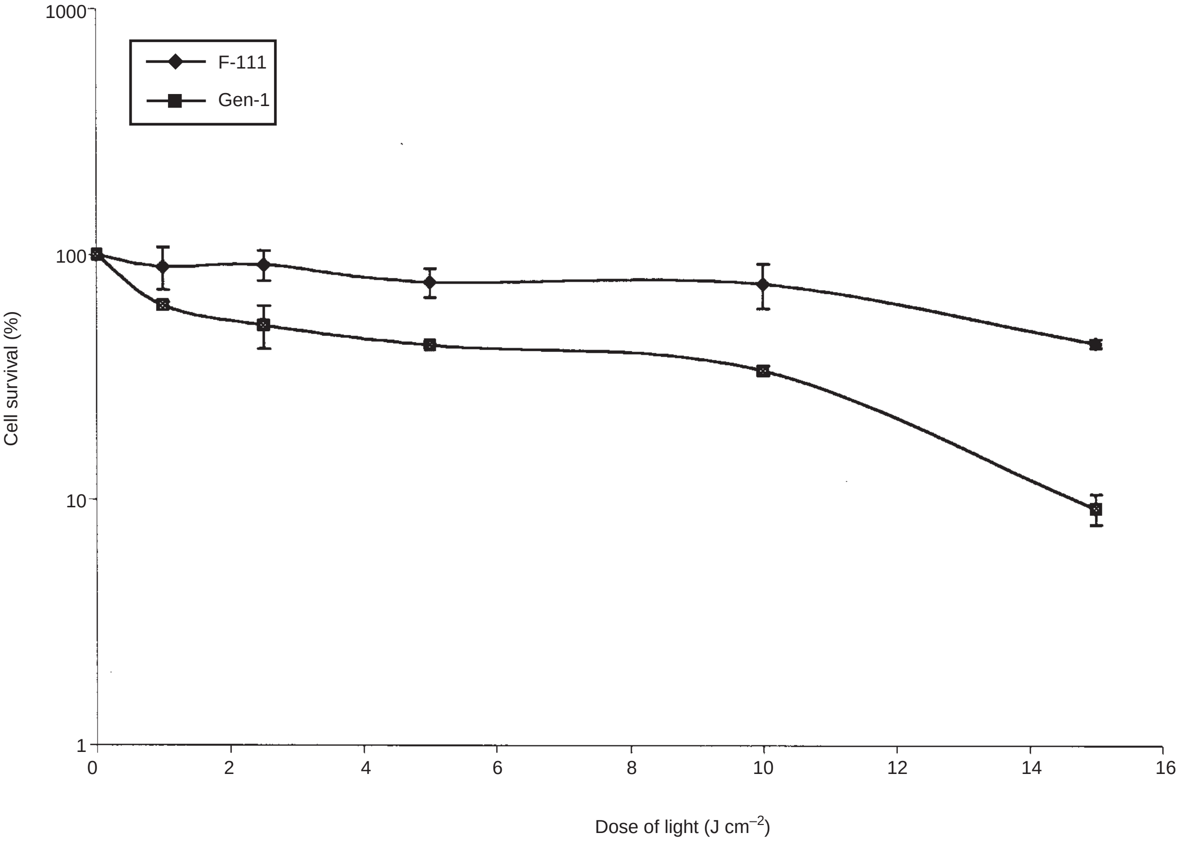

**Figure 6** Proliferative capacity of rat fibroblasts incubated for 15 h with 3 mM ALA without added inducer and then exposed to graded doses of photoactivating light, as assessed by thymidine incorporation (mean ± s.d. of three experiments). The difference between the photoradiation survival curves of immortalized Fisher rat fibroblasts (F-111 cells) and F-111 cells transfected with polyomavirus early region middle T antigen oncogene (Gen-1) is highly significant ($P$ < 0.0002, two-way ANOVA)

 © Cancer Research Campaign 1999

resistance gene). Cells expressing the c-*myc* oncogene, which can establish fibroblasts in culture but is unable to transform them to full malignancy (Land et al, 1983), showed high levels of ALA-induced fluorescence also.

### Correlation between ALA phenotype and malignant potential

The malignant potential of the various mouse cell lines used in the above experiments was evaluated by (a) anchorage independent colony formation assays in soft agar, and (b) tumour formation following subcutaneous injection of the cells into histocompatible mice. None of the immortalized fibroblast cell lines formed colonies in soft agar or tumours in syngeneic mice. All of the oncogene-transfected mouse fibroblasts and Balb/c fibrosarcomas formed colonies in soft agar, but only the fibrosarcoma cell lines formed tumours in syngeneic mice. There was complete correlation between the malignant ALA phenotype and anchorage independent colony formation in soft agar.

### Quantitation of ALA-induced photosensitization by $^3$H-thymidine incorporation

F-111 and Gen-1 cell suspensions that had been incubated with ALA were exposed to graded doses of photoactivating light, and then incubated with $^3$H-thymidine. As might be expected if the intensity of the ALA-induced PpIX photosensitization is related to the intensity of the PpIX fluorescence, thymidine incorporation was more strongly inhibited in the Gen-1 oncogene-transformed cells than in the F-111 parental cell line (Figure 6).

### ALA-induced PpIX photosensitization in vivo

Balb/c fibrosarcoma cell suspensions were injected s.c. into the lower left flank of Balb/c mice. When the tumours had grown to 2–4 mm in diameter, doses of ALA ranging from 100 to 300 mg per kg of body weight were injected i.p., and the tumour and adjacent skin exposed to photoactivating light (216 J cm$^{-2}$) 3 h later. All of the tumours became highly necrotic, although none were completely eradicated. In contrast, the adjacent skin that had received a similar dose of light showed a much milder response and quickly recovered.

### DISCUSSION

When exposed to ALA in vivo, murine fibrosarcomas developed stronger PpIX fluorescence and became more strongly photosensitized than adjacent fibrous connective tissues. Our fibroblast model reproduced this relationship in tissue culture. Normal embryonic fibroblasts, a selection of immortalized fibroblast cell lines, and immortalized fibroblasts transfected with a drug resistance gene developed PpIX fluorescence intensities that were relatively low and statistically indistinguishable, while fibrosarcoma cells and immortalized fibroblasts transfected with specific oncogenes showed significantly greater fluorescence. This difference in fluorescence was visualized by fluorescence microscopy (data not shown), and was quantitated by spectrophotofluorometry of confluent cell monolayers (Figures 1 and 2), soluble extracts of cells and supernatants (Figure 3), and fluorescence flow cytometry of single cell suspensions (Figures 4 and 5). There was a corresponding difference in ALA-induced photosensitization, with malignant fibroblasts being significantly more photosensitive than non-malignant fibroblasts that had been incubated with ALA under similar conditions (Figure 6).

The ALA phenotypes of F-111 rat fibroblasts transfected with the G418-resistance gene (cell lines Fneo-1v and Fneo-1w) were indistinguishable from the phenotype of the parental F-111 cell line, an indication that the process of transfection as such produced no detectable effect upon the response of F-111 cells to exogenous ALA (Figure 5). However, even the low levels of oncoproteins expressed by F-111 cells transfected with inducible polyomavirus oncogenes which had not been induced (Raptis et al, 1985) were associated with significant increases in ALA-induced fluorescence and photosensitization (Figures 2 and 5). This finding is consistent with clinical observations that the malignant ALA phenotype is expressed early during the process of malignant transformation, as indicated by its presence in premalignant lesions such as actinic keratosis.

Chemical extraction of cells and supernatants remains the ‘gold standard’ for quantitating ALA-induced PpIX. This technique can measure both monomeric (fluorescent) and aggregated (non-fluorescent) PpIX and can distinguish between different types of endogenous porphyrins, but it can not detect subpopulations of cells that have different ALA-induced phenotypes. Spectrophotofluorometry of cell monolayers or cell suspensions can measure the monomeric prophyrins in cells and/or supernatants and can distinguish between various types of prophyrins, but it can not detect subpopulations. Fluorescence flow cytometry readily distinguishes subpopulations of cells that have different ALA phenotypes, but it can not identify the type of porphyrin involved or measure porphyrin(s) in the supernatant. All three techniques are useful, but they are not interchangeable.

Comparison of ALA-induced fluorescence and/or photosensitization in normal, immortalized, oncogene-transfected, and malignant fibroblasts may provide a simple, rapid and quantitative technique for detecting early events during the process of malignant transformation. It may permit detection, enumeration and collection of cells at early stages of transformation by flow cytometry, and rapid quantitative comparison of the transforming efficiency of ionizing and non-ionizing radiations, chemical carcinogens, oncogenic viruses and oncogene-bearing plasmids under various conditions. It may also permit the rapid evaluation of putative promoters and/or protective agents, and rapid quantitation of the effect of oncogene activation or of blocking the expression of specific oncogenes or anti-oncogenes. Finally, our data indicate that fibrosarcoma cells can be killed preferentially by ALA-PDT. Since human fibrosarcomas often are quite resistant to both chemotherapy and radiotherapy (Yang et al, 1993), ALA-PDT might be considered as an adjunct to more standard forms of therapy.

### ACKNOWLEDGEMENTS

We thank Mr Lloyd Kennedy and Ms Pamela Bandy-Dafoe for their technical assistance, Dr R Baserga of Jefferson University, Philadelphia, PA, USA, for Balb/c 3T3 oncogene transfectants, Dr U Rapp of the University of Wurzberg, Germany, for NIH 3T3 oncogene transfectants, and Dr T Yamashita for rat fibroblasts transfected with v-*abl*. This research was supported by DRAXIS Pharmaceuticals, Inc., Toronto, Canada, and by Cancer Care Ontario.

© Cancer Research Campaign 1999 

### REFERENCES

Baserga R, Reiss K, Alder H, Pietrzkowski Z and Surmacz E (1992) Inhibition of cell cycle progression by antisense oligodeoxynucleotides. *Ann NY Acad Sci* **660**: 64–69

Campbell DL, Fisher ME, Johnson JG, Rossi FM, Campling BG, Pottier RH and Kennedy JC (1996*a*) Flow cytometric technique for quantitating cytotoxic response to photodynamic therapy. *Photochem Photobiol* **63**: 111–116

Campbell DL, Gudgin-Dickson EF, Forkert PG, Pottier RH and Kennedy JC (1996*b*) Detection of early stages of carcinogenesis in adenomas of murine lung by 5-aminolevulinic acid-induced protoporphyrin IX fluorescence. *Photochem Photobiol* **64**: 676–682

Golub AL, Gudgin-Dickson EF, Kennedy JC, Marcus SL, Park Y and Pottier RH (1998) The monitoring of ALA-induced protoporphyrin IX accumulation and clearance in patients with skin lesions by in vivo surface-detected fluorescence spectroscopy. *Lasers Med Sci* (in press)

Kennedy JC and Pottier RH (1992) Endogenous protoporphyrin IX, a clinically useful photosensitizer for photodynamic therapy. *J Photochem Photobiol B*: *Biol* **14**: 275–292.

Kennedy JC, Pottier RH and Pross DC (1990) Photodynamic therapy with endogenous protoporphyrin IX: basic principles and present clinical experience. *J Photochem Photobiol B*: *Biol* **6**: 143–148

Kennedy JC, Marcus SL and Pottier RH (1996) Photodynamic therapy (PDT) and photodiagnosis (PD) using endogenous photosensitization induced by 5-aminolevulinic acid (ALA): mechanisms and clinical results. *J Clin Laser Med Surg* **14**: 289–304

Kiss Z, Rapp UR, Pettit GR and Anderson WB (1991) Phorbol ester and bryostatin differentially regulate the hydrolysis of phosphatidylethanolamine in Ha-*ras*- and *raf*-oncogene-transformed NIH 3T3 cells. *Biochem J* **276** (Pt 2): 505–509

Land H, Parada LF and Weinberg RA (1983) Tumorigenic conversion of primary embryo fibroblasts requires at least two cooperating oncogenes. *Nature* **304**: 596–602

Loo D and Cotman CW (1998) Primary and extended culture of embryonic mouse cells. In *Cell Biology*: *a Laboratory Manual*, Celis JE (ed), pp. 65–72. Academic Press: London

Peng Q, Berg K, Moan J, Kongshaug M and Nesland JM (1997*a*) 5-aminolevulinic acid-based photodynamic therapy: principles and experimental research. *Photochem Photobiol* **65**: 235–251

Peng Q, Warloe T, Berg K, Moan J, Kongshaug M, Giercksky KE and Nesland JM (1997b) 5-aminolevulinic acid-based photodynamic therapy: clinical research and future challenges. *Cancer* **79**: 2282–2308

Raptis L, Lafrom H and Benjamin TL (1985) Regulation of cellular phenotype and expression of polyomavirus middle T antigen in rat fibroblasts. *Mol Cell Biol* **5**: 2476–2486

Roy BN, Van Vugt DA, Weagle GE, Pottier RH and Reid RL (1997) Effect of 5-aminolevulinic acid dose and estrogen on protoporphyrin IX concentrations in the rat uterus. *J Soc Gynecol Invest* **4**: 40–46

Yamashita T, Kato H and Fujinaga K (1988) Conditional immortalization and/or transformation of rat cells carrying v-*abl* or EJ*ras* oncogene in the presence or absence of glucocorticoid hormone. *Int J Cancer* **42**: 930–938

Yang JC, Glastein EJ, Rosenberg SA and Antman KH (1993) Sarcoma of soft tissues. In *Cancer*: *Principles and Practice of Oncology*, DeVita VT Jr, Hellman S and Rosenberg SA (ed), pp. 1436–1488. JB Lippincott: Philadelphia

*© Cancer Research Campaign 1999*
